# Intraductal tubulopapillary neoplasms with rupture of the distal main pancreatic duct: a case report

**DOI:** 10.1186/s40792-020-00972-0

**Published:** 2020-08-14

**Authors:** Yuji Shimizu, Ryo Ashida, Teiichi Sugiura, Yukiyasu Okamura, Takaaki Ito, Yusuke Yamamoto, Katsuhisa Ohgi, Keiko Sasaki, Katsuhiko Uesaka

**Affiliations:** 1 Division of Hepato-Biliary-Pancreatic Surgery, Shizuoka Cancer Center, 1007 Shimo-Nagakubo, Sunto-Nagaizumi, Shizuoka, 411-8777 Japan; 2 Division of Pathology, Shizuoka Cancer Center, 1007 Shimo-Nagakubo, Sunto-Nagaizumi, Shizuoka, 411-8777 Japan

**Keywords:** Intraductal tubulopapillary neoplasm, Pancreas, Rupture

## Abstract

**Background:**

Intraductal tubulopapillary neoplasm (ITPN) is a rare and newly described entity defined as an intraductal, grossly visible, tubule-forming epithelial neoplasm with high-grade dysplasia and ductal differentiation without overt production of mucin. Because of its rarity, the clinical and molecular aspects of ITPN have not been fully investigated.

**Case presentation:**

A 73-year-old woman presented to a local hospital with epigastric discomfort and pain. Abdominal multidetector-row computed tomography (MDCT) revealed a 2.5-cm hypovascular tumor in the pancreatic body with distal pancreatic duct dilatation and a slightly low-density area spreading over the ventral side of the pancreatic body. Endoscopic ultrasonography and fine-needle biopsy of the tumor revealed adenocarcinoma of the pancreas. She was referred to our hospital 2 months later. MDCT performed at our hospital showed no significant change in the tumor size or pancreatic duct dilatation. However, the low-density area at the ventral side of the pancreas had shrunk; therefore, this finding was considered to have been an inflammatory change. Under a preoperative diagnosis of resectable pancreatic ductal adenocarcinoma, distal pancreatectomy was performed. The final diagnosis was ITPN with associated invasive carcinoma. Macroscopically and microscopically, the main pancreatic duct (MPD) had ruptured at the distal side of the tumor, and the fistula connected the MPD and extrapancreatic scar tissue.

**Conclusions:**

ITPN with rupture of the pancreatic duct is extremely rare. In the present case, a sudden increase in the pancreatic duct internal pressure or acute inflammation likely caused the rupture of the MPD.

## Introduction

Intraductal tubulopapillary neoplasm (ITPN) was first described in 2009 [1] and formally introduced in the 2010 World Health Organization classification [2] as a distinct entity among premalignant epithelial tumors of the pancreas. ITPN is defined as an intraductal, grossly visible, tubule-forming epithelial neoplasm with high-grade dysplasia and ductal differentiation without overt production of mucin [2].

ITPNs are estimated to account for < 1% of all pancreatic exocrine tumors and 3% of all pancreatic intraductal neoplasms [1]. Because of the rarity and relatively recent introduction of ITPNs, the clinical and molecular aspects of ITPNs remain unclear. An ITPN usually appears as a solid mass obstructing the main pancreatic duct (MPD), thereby causing distal ductal dilatation [3]. However, ITPN-associated rupture of the MPD is extremely rare, and only one case has been reported to date [4].

We herein report a case of a resected ITPN that was associated with rupture of the MPD.

## Case report

**Fig. 1:**
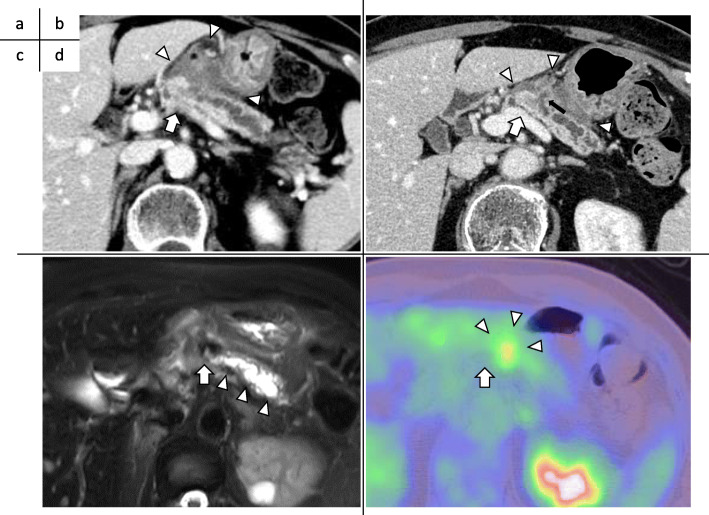
Imaging findings. **a** Abdominal multidetector-row computed tomography (MDCT) at the referral hospital. A hypovascular tumor was present in the body of the pancreas (arrow), and the distal pancreatic duct was dilated. In addition, a slightly low-density area (LDA) was found to be spread over the ventral side of the pancreas (arrowhead) and touching the stomach. **b** MDCT images obtained 2 months later at our hospital. There was no significant change in the tumor size (arrow) or pancreatic duct dilatation, but the LDA at the ventral side of the pancreas had shrunk (arrowhead). The two-tone duct sign and cork-of-wine-bottle sign were observed (arrow). Retrospective examination revealed findings that seemed to indicate the rupture of the main pancreatic duct (MPD) and the formation of a fistula extending outside of the pancreas (black arrow). **c** T2-weighted magnetic resonance image taken at our hospital. Constriction of the MPD (arrow) and distal MPD dilatation (arrowhead) was observed, but the tumor was unclear. **d** Positron emission tomography-computed tomography taken at the referral hospital. Weak accumulation of fluorodeoxyglucose was present at the ventral side of the pancreas, corresponding to the LDA on MDCT (arrowhead); however, no apparent accumulation was noted in the tumor (arrow)

A 73-year-old woman presented to a local hospital with a 4-month history of persistent epigastric discomfort and pain. Abdominal multidetector-row computed tomography (MDCT) revealed a 2.5-cm hypovascular tumor in the pancreatic body with distal pancreatic ductal dilatation. A slightly low-density area (LDA) was found to be spread over the ventral side of the pancreatic body and touching the stomach (Fig. 1a). Endoscopic ultrasonography and fine-needle biopsy of the tumor revealed adenocarcinoma of the pancreas. The patient was therefore referred to our hospital for further examination and treatment 2 months later.

Upon presentation, the patient’s serum levels of carcinoembryonic antigen (1.1 ng/mL), carbohydrate antigen 19-9 (12 U/mL), duke pancreatic monoclonal antigen type 2 (< 25 U/mL), and s-pancreas antigen-1 (9.0 U/mL) were within the normal ranges. MDCT was performed again at our institution, along with magnetic resonance imaging (MRI). MDCT showed no significant change in the tumor size or pancreatic duct dilatation, but the LDA at the ventral side of the pancreas had shrunk (Fig. 1b). MRI showed constriction of the MPD and distal pancreatic ductal dilatation, but the tumor was unclear (Fig. 1c). Positron emission tomography-computed tomography showed weak accumulation of fluorodeoxyglucose at the ventral side of the pancreas, corresponding to the LDA on MDCT (Fig. 1d). However, no apparent accumulation was noted in the tumor (Fig. 1d). Distal pancreatectomy was planned under a preoperative diagnosis of resectable pancreatic ductal adenocarcinoma (PDAC) of the pancreatic body. The LDA at the ventral side of the pancreas shrank within a short period, suggesting that this finding was a pancreatitis-induced inflammatory change.

**Fig. 2:**
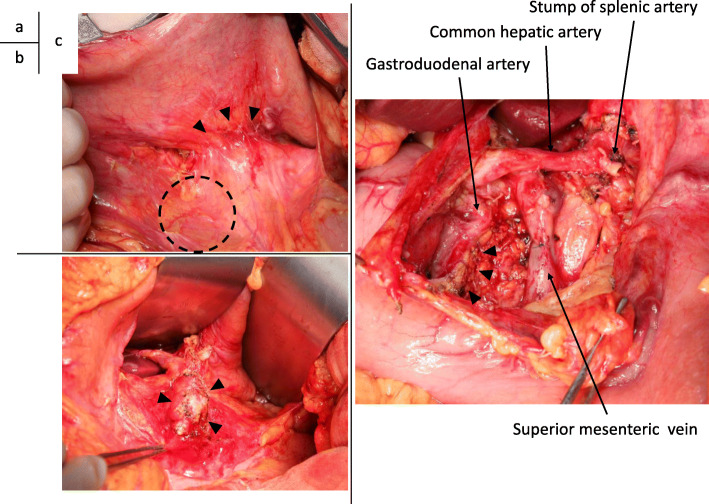
Intraoperative findings. **a** At laparotomy, the tumor was found within the pancreatic body (dashed circle). No serosal or vascular invasion was present. On the tail side of the tumor, severe adhesion was observed between the pancreas and the stomach (arrowhead). **b** Frontal view of the pancreas after partial resection of the serosa of the stomach. The intraoperative frozen section examination confirmed the lack of any cancer cells on the detached surface (arrowhead). **c** The pancreas was divided at the left edge of the gastroduodenal artery (arrowhead). Distal pancreatectomy with radical lymphadenectomy and splenectomy was then completed

During laparotomy, the tumor was found within the pancreatic body. No serosal or vascular invasion was present. No ascites, peritoneal dissemination, or liver metastasis were observed. Peritoneal lavage cytology was negative for cancer. On the tail side of the tumor, severe adhesion was present between the pancreas and stomach (Fig. 2a). This adhesion was very difficult to dissect, and combined resection of the stomach serosa was therefore performed. An intraoperative frozen section examination of the dissected margin of the stomach serosa was negative for cancer (Fig. 2b). The pancreas was divided at the left edge of the gastroduodenal artery (Fig. 2c). Frozen section examination of the pancreatic stump was negative for cancer. Distal pancreatectomy with radical lymphadenectomy and splenectomy was then completed (Fig. 2c).

**Fig. 3:**
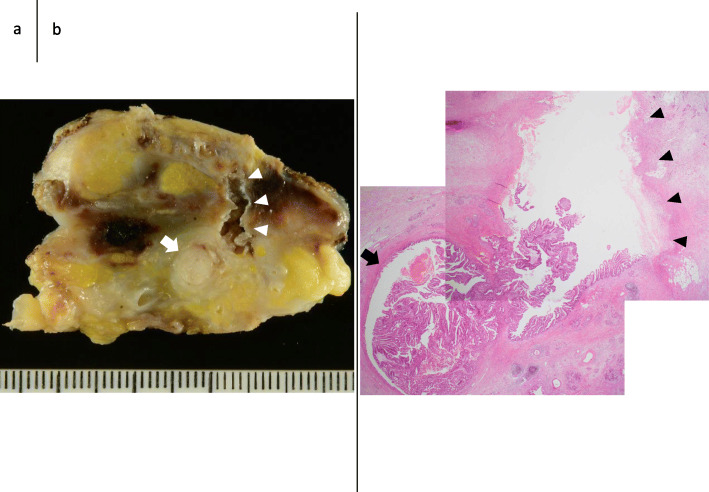
Macroscopic findings. **a** Enlarged view of a section of the resected specimen after formalin fixation containing the main tumor. **b** A loupe view of the same section. A tubulopapillary tumor measuring 37 × 25 mm was found within the main pancreatic duct (arrow), and a fistula connecting the main pancreatic duct to the extrapancreatic scar tissue had formed (arrowhead)

**Fig. 4:**
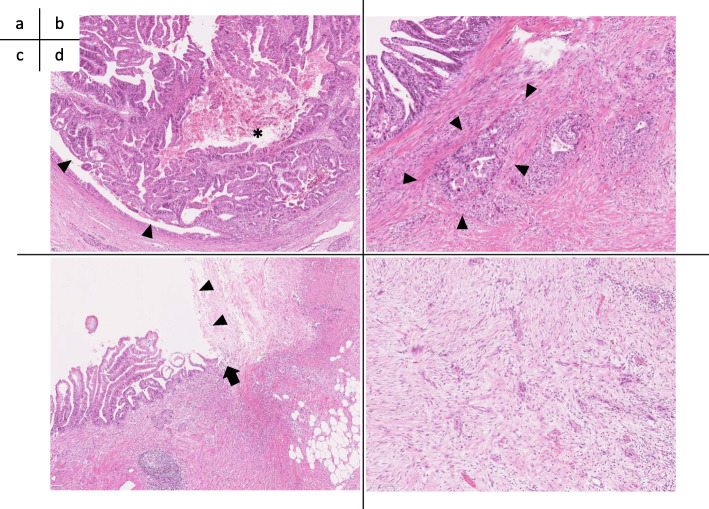
Microscopic findings. **a** Microscopic findings of the tumor in the main pancreatic duct (MPD). The tumor showed a tubulopapillary growth pattern (arrowhead), and necrosis was observed (asterisk) (hematoxylin and eosin [H&E]; original magnification, × 50). **b** The tumor had invaded the stroma around the MPD (arrowhead) (H&E; original magnification, ×100). **c** The pancreatic duct was ruptured at the tail side of the tumor (arrow), and the fistula connected the MPD and the extrapancreatic scar tissue (arrowhead) (H&E; original magnification, × 50). **d** Microscopic findings of the tissues outside of the pancreas. Granulation and fibrosis were observed, and cancer cells were scattered (H&E; original magnification, × 100)

The macroscopic findings of the resected specimen are shown in Fig. 3. A solid tumor measuring 37 × 25 mm was found within the MPD, and a fistula connecting the MPD to extrapancreatic scar tissue had formed (Fig. 3a, b). Microscopically, the tumor showed intraductal tubulopapillary growth with necrosis (Fig. 4a) with invasion into the stroma around the MPD (Fig. 4b). No mucin production was present. The MPD had ruptured at the distal side of the tumor (Fig. 4c), and the fistula connected the MPD and extrapancreatic scar tissue (Fig. 4c). Granulation and fibrosis were observed in the extrapancreatic tissues, and scattered cancer cells were observed (Fig. 4d). No vascular invasion, perineural infiltration, or lymph node metastasis was present. The immunohistochemical staining results were MUC1 (+), MUC2 (−), MUC5AC (−), and MUC6 (+), resulting in a final diagnosis of ITPN with associated invasive carcinoma, moderately differentiated adenocarcinoma, pT2, pN0, pM0, pStage IB according to the Union for International Cancer Control TNM classification (8th edition) [5]. The invasive component had spread 30 mm along the MPD. The pancreatic cut margin was negative for cancer. Administration of S-1 was performed as adjuvant chemotherapy [6]. The patient was alive at the time of this writing (10 months postoperatively) without recurrence.

## Discussion

We encountered a case of surgically resected ITPN that had caused MPD rupture. Macroscopically and microscopically, the tumor showed tubulopapillary growth within the MPD, and at the distal side of the tumor, a fistula connected the MPD to the extrapancreatic scar tissue.

The main characteristics of ITPN are a solid nodular tumor macroscopically obstructing dilated ducts, no visible secreted mucin; tubulopapillary growth; uniform high-grade atypia throughout the neoplasm; easily recognizable necrotic foci; ductal differentiation as indicated by MUC1 and CK7 expression; absence of acinar differentiation as indicated by the absence of trypsin; absence of MUC2, MUC5AC, and fascine; and absence of *KRAS* and *BRAF* mutations [7]. Most of these important histopathological characteristics of ITPN were present in our case. The characteristic imaging findings of ITPN are a two-tone duct sign and cork-of-wine-bottle sign on MRI or endoscopic retrograde cholangiopancreatography [8]. In the present case, although these findings were not obvious on MRI, MDCT showed similar findings (Fig. 1b). In addition, upon retrospective examination, findings that seemed to indicate MPD rupture and the formation of a fistula extending outside of the pancreas were present on MDCT (Fig. 1b).

Regarding treatment, several authors have recommended radical surgery for resectable ITPN, just as for PDAC [9, 10]. The prognosis of ITPN is considered to be better than that of intraductal papillary mucinous carcinoma or PDAC [9, 11]; however, there have been no comprehensive reports on the efficacy of chemotherapy for ITPN. In the present case, the final diagnosis was ITPN with associated invasive carcinoma. The MPD had ruptured and cancer cells were scattered toward the extrapancreatic tissues. However, intraoperative lavage cytology was negative for cancer, and curative resection was achieved. We considered that additional treatment was needed because of the risk of peritoneal dissemination recurrence due to MPD rupture and potential leakage of tumor cells despite the fact that the intraoperative lavage cytology was negative. Although its efficacy was unclear, S-1 was administered as adjuvant chemotherapy because this is the standard treatment for PDAC in Japan [6].

A literature review of 73 cases of ITPN showed that approximately 90% of the cases were associated with MPD dilatation [12]. However, the rupture of the MPD was extremely rare. A PubMed search using the key words “ITPN” and “rupture” revealed only one case, in which an ITPN with the formation of gigantic pancreatic cysts ruptured and caused acute peritonitis [4]. The authors considered that solid ITPN formation in the MPD had obstructed the flow of pancreatic juice and increased the internal pressure of the distal pancreatic duct, leading to the development of gigantic pancreatic cysts and MPD rupture [4].

**Fig. 5:**
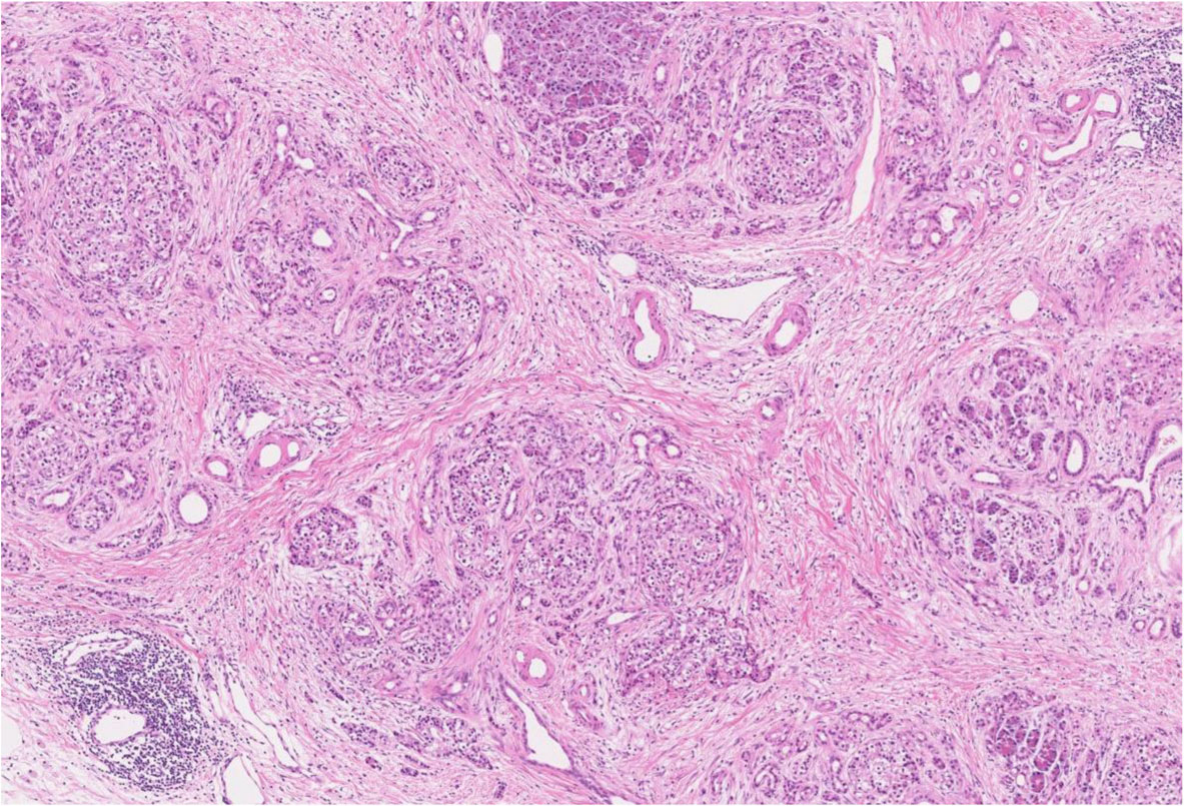
Microscopic findings of pancreatic parenchyma on the section of the tail side of the tumor. Atrophy of acinar cells and fibrosis were localized, and the acinar cells were relatively retained (hematoxylin and eosin; original magnification, × 50)

Regarding the mechanisms underlying MPD rupture, differences in the tumor nature between ITPN and PDAC may be important. PDAC is morphologically characterized by an intense fibrotic process called a desmoplastic reaction. Therefore, PDAC is often associated with chronic obstructive pancreatitis with extensive fibrosis and atrophy of acinar cells in the pancreatic parenchyma on the tail side of the tumor [13, 14]. In PDAC, pancreatic duct stenosis develops slowly, and the internal pressure within the pancreatic duct rises gradually. At the same time, the pancreatic parenchyma becomes fibrotic and hardens. Therefore, the pancreatic duct may not easily rupture outside the pancreas. In contrast, in the present case, atrophy of acinar cells and fibrosis were localized at the tail side of the tumor, and the acinar cells were relatively retained (Fig. 5). Based on these findings, we speculated that the MPD rupture was caused by the following three mechanisms: the tumor gradually grew within the MPD and suddenly obstructed its lumen, causing a rapid rise in the internal pressure of the pancreatic duct and acute inflammation; the remaining pancreatic exocrine function distal to the tumor caused pancreatic juice to be retained, contributing to the rapid increase in the MPD pressure; and the pancreatic parenchyma distal to the tumor was softer and less pressure-resistant than that of PDAC because the fibrosis was localized.

However, considering that perforations are extremely rare, patient-specific factors such as anatomical features and tissue strength are likely involved in such cases. Unfortunately, no pre-perforation images were available in our patient, and elaborate discussion was difficult. Further case reports are desired.

In conclusion, we encountered an extremely rare case of ITPN with the rupture of the MPD. A sudden increase in pancreatic duct internal pressure or acute inflammation likely caused the MPD rupture.

## Data Availability

The authors declare that all the data in this article are available within the article.

## Abbreviations

ITPN: Intraductal tubulopapillary neoplasm
MPD: Main pancreatic duct
MDCT: Multidetector-row computed tomography
LDA: Low-density area
MRI: Magnetic resonance imaging
PDAC: Pancreatic ductal adenocarcinoma
